# PCL strut-like scaffolds appear superior to gyroid in terms of bone regeneration within a long bone large defect: An *in silico* study

**DOI:** 10.3389/fbioe.2022.995266

**Published:** 2022-09-23

**Authors:** Mahdi Jaber, Patrina S. P. Poh, Georg N. Duda, Sara Checa

**Affiliations:** ^1^ Berlin Institute of Health at Charité – Universitätsmedizin Berlin, Julius Wolff Institute, Berlin, Germany; ^2^ Berlin-Brandenburg School for Regenerative Therapies, Berlin, Germany; ^3^ BIH Center for Regenerative Therapies, Berlin, Germany

**Keywords:** mechano-biology, bone defect healing, 3D-printed scaffold design, bone tissue engineering, gyroid, TPMS

## Abstract

The treatment of large bone defects represents a major clinical challenge. 3D printed scaffolds appear as a promising strategy to support bone defect regeneration. The 3D design of such scaffolds impacts the healing path and thus defect regeneration potential. Among others, scaffold architecture has been shown to influence the healing outcome. Gyroid architecture, characterized by a zero mean surface curvature, has been discussed as a promising scaffold design for bone regeneration. However, whether gyroid scaffolds are favourable for bone regeneration in large bone defects over traditional strut-like architecture scaffolds remains unknown. Therefore, the aim of this study was to investigate whether gyroid scaffolds present advantages over more traditional strut-like scaffolds in terms of their bone regeneration potential. Validated bone defect regeneration principles were applied in an *in silico* modeling approach that allows to predict bone formation in defect regeneration. Towards this aim, the mechano-biological bone regeneration principles were adapted to allow simulating bone regeneration within both gyroid and strut-like scaffolds. We found that the large surface curvatures of the gyroid scaffold led to a slower tissue formation dynamic and conclusively reduced bone regeneration. The initial claim, that an overall reduced zero mean surface curvature would enhance bone formation, could not be confirmed. The here presented approach illustrates the potential of *in silico* tools to evaluate in pre-clinical studies scaffold designs and eventually lead to optimized architectures of 3D printed implants for bone regeneration.

## 1 Introduction

The treatment of large bone defects represents a major clinical challenge. Current treatment strategies, such as autologous bone grafting, although clinically successful, have substantial drawbacks and limitations; e.g., the need for additional surgical access to the donor site, limited availability of adequate bone to fill a critical-sized defect and subsequent morbidity of the donor site (Roddy et al., 2018).

Porous scaffolds made from biomaterials and printed in 3D to match the patient-specific defect dimensions have a high potential to overcome the above mentioned limitations and solve the medical need in treating critical size large bone defects (Pobloth et al., 2018). Different scaffold designs have been experimentally tested, both *in vitro* and in pre-clinical studies (Cobos et al., 2000; Reichert et al., 2012; Lovati et al., 2016; Shah et al., 2016; Reznikov et al., 2019). It has been shown that, among others, scaffold material, pore size, porosity, permeability and overall stiffness influence the healing outcome (Schlichting et al., 2008; Mitsak et al., 2011; Pobloth et al., 2018; Wei et al., 2020).

Recently, triply periodic minimal surfaces (TPMS) scaffolds have gained high interest in tissue engineering. They are thought to resemble the bone microarchitecture due to their biomimetic geometry (Dong and Zhao, 2021). TPMS scaffolds are characterized by a mean surface curvature of zero (Jinnai et al., 2002), similar to what has been reported for trabecular bone (Abueidda et al., 2019). In addition, the surface area to volume ratio of TPMS scaffolds is relatively high compared to other scaffold designs (Lu et al., 2020; du Plessis et al., 2018). This higher surface area of TPMS scaffolds has been shown to contribute to enhanced cell adhesion, support migration on such surfaces, and enable proliferation (Yoo, 2014; Vijayavenkataraman et al., 2018).

TPMS can adopt different configurations. Among them, gyroid is the most popular for creating architectures with robust mechanical performance (Abueidda et al., 2019). Gyroid has no planes of reflection symmetry and no straight line segments lying on its surface (Karcher, 1989; Große-Brauckmann and Wohlgemuth, 1996). Interestingly, gyroid architectures are found in nature, e.g., in mitochondria’s inner membranes (Cui et al., 2020) and butterfly wings (Michielsen and Stavenga, 2008). Moreover, the continuous curvature of their struts has been suggested to be beneficial avoiding concentration of mechanical stresses (Yánez et al., 2020). Gyroid scaffolds were shown to exhibit high permeability when compared to other TPMS scaffolds (Castro et al., 2019a; Santos et al., 2020). In addition, compared to other TPMS scaffolds, gyroid scaffolds show high stiffness (Yang et al., 2019), crucial for maintaining a stable mechanical environment, which makes them especially interesting for bone regeneration applications (Glatt et al., 2017).

Although numerous experimental and numerical studies have been performed on scaffolds with gyroid architecture in recent years, most of these studies have been limited to analysing their mechanical properties (Melchels et al., 2010b; Dalaq et al., 2016; Yánez et al., 2018; Castro et al., 2019b; Alizadeh-Osgouei et al., 2021). For example, several studies have investigated the influence of the pore size of gyroid scaffolds on their overall mechanical properties (Ma et al., 2019; Alizadeh-Osgouei et al., 2021; Caiazzo et al., 2021). Only a few *in vitro* studies have investigated cellular behaviour within gyroid scaffolds. Melchels et al. (2010a) showed that their open architecture facilitates cell infiltration into the scaffold, which has been attributed to the zero-mean curvature (Rajagopalan and Robb, 2006). In addition, very few *in vivo* studies have investigated the bone regeneration potential of gyroid scaffolds. Kelly et al. (2021) investigated the potential of gyroid scaffolds in a rat femoral defect; however, they didn’t compare its healing outcome to a different scaffold design. Van hede et al. (2022) showed that gyroid scaffolds lead to slightly more bone formation when compared to traditional strut-like scaffolds within a skull bone defect. However, bone regeneration within a skull defect occurs under highly reduced mechanical conditions. So far, whether gyroid scaffolds present advantages over traditional scaffolds (e.g., strut-like scaffolds) in large long bone defects, remains unknown.

Ongoing research is moving towards the use of approaches that satisfy the 3Rs principle, i.e., reduction, replacement and refinement (Viceconti and Dall’Ara, 2019). *In silico* approaches offer the unique opportunity to investigate virtually, potential mechanisms behind biological processes and even to investigate interactions that are difficult or even impossible to measure experimentally. In addition, they allow testing of potential treatment strategies, reducing the need for pre-clinical experiments (Viceconti and Dall’Ara, 2019; Jean-Quartier et al., 2018). In the field of bone regeneration, numerous computer models have been developed and validated for their potential to predict regeneration in uneventful bone healing conditions (Byrne et al., 2011; Checa et al., 2011; Vetter et al., 2012; Repp et al., 2015; Borgiani et al., 2019). Recently, these models have been further developed and validated in their potential to predict bone regeneration within scaffolds (Perier-Metz et al., 2020; Perier-Metz et al., 2022). However, they have never investigated bone regeneration within gyroid scaffolds.

The aim of this study was to investigate whether gyroid scaffolds would be favourable for bone regeneration over more traditional scaffolds, i.e., a strut-like scaffold, applying principles of bone formation in an *in silico* modelling approach. The bone regeneration potential of a gyroid scaffold as well as the influence of mechanical cues and cellular dynamics throughout the regeneration was compared with that of a more traditional strut-like design to assess the suggested benefits of a gyroid architecture compared with more traditional designs. We hypothesized that a gyroid scaffold design would promote cellular activities involved in bone regeneration and therefore show enhanced bone regeneration compared to a strut-like configuration, as predicted by an *in silico* model.

## 2 Materials and methods

### 2.1 *In silico* bone regeneration model

A previously described bone regeneration computer model able to explain experimentally observed scaffold-supported bone regeneration in a large bone defect (Perier-Metz et al., 2020; Perier-Metz et al., 2022) was adapted to investigate bone regeneration within gyroid scaffolds. The computer model combined finite element (FE) analysis to determine the mechanical environment within the scaffold, and an agent-based model (ABM) describing the biological processes taking place during bone regeneration (Perier-Metz et al., 2020). Scaffolds were virtually inserted into a critical size large bone defect in the rat femur to mimic an *in vivo* experimental setup, so that the model could be used to inform a potential future pre-clinical study. This experimental setup has been previously used to investigate bone regeneration within large bone defects both *in vivo* (Schwarz et al., 2013) and *in silico* (Borgiani et al., 2021).

#### 2.1.1 Finite element model

**FIGURE 1:**
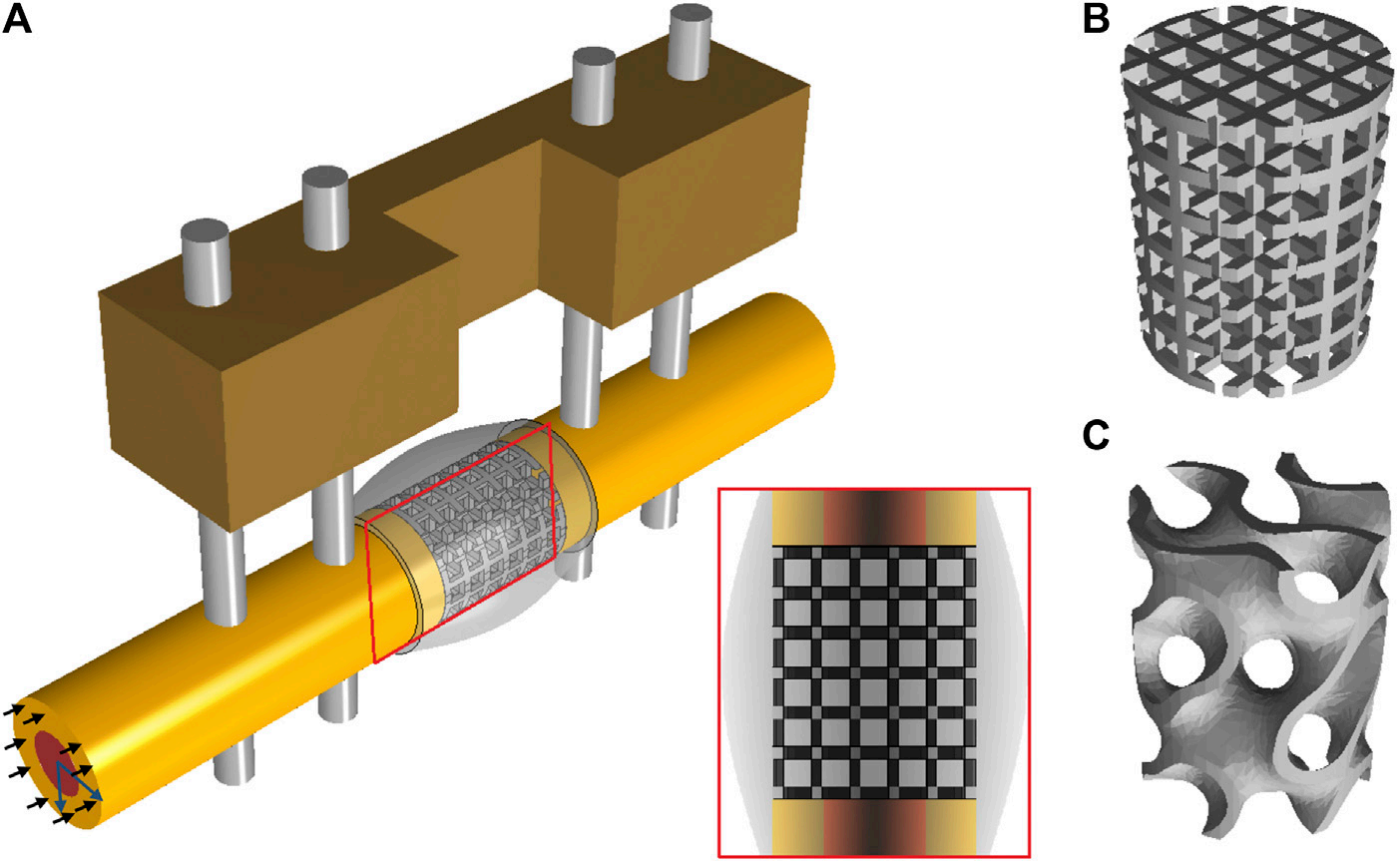
**(A)** CAD model simulating a large bone defect in the rat femur stabilized with an external fixator. In the defect a strut-like **(B)** or a gyroid **(C)** scaffold was virtually inserted. The black arrows represent the compressive loads and the blue arrows represent the tangential loads inducing bending.

A 3D finite element model was created to assess the mechanical environment inside the large bone defect and scaffold pores. The FE model was developed in ABAQUS/Standard 2019 (Simulia, Dassault Systemes). The model simulated a large bone defect in the rat femur stabilized with an external fixator, following previous experimental studies (Schwarz et al., 2013). The computer model included the cortical bone, the marrow cavity, the external fixator and the scaffold fitted in the defect region surrounded by a callus. The geometry of the bone was modelled as a hollow cylinder representing the cortical bone and an internal marrow cavity. The large bone defect was replicated by opening a 5-mm-wide gap in the middle of the bone. The geometry of the model is based on a previous computer model (Borgiani et al., 2021) that approximated the callus dimensions based on histological data (Schwarz et al., 2013). To better assess the regeneration potential of the gyroid scaffold, scaffolds with two different architectures and with the same overall geometry (height 5 mm and radius 2 mm) and porosity (79%) were developed: one having a more traditional strut-like architecture and the other having a gyroid configuration (Figure 1). The strut-like scaffold (Figure 1B) was directly modelled using ABAQUS/Standard 2019 with a pore size of 0.54 mm, resulting in a surface area to volume ratio of 3.74. The gyroid scaffold (Figure 1C) was created in Rhinoceros 3D (Robert McNeel & Associates) with a pore size of 0.56 mm and a surface area to volume ratio of 2.35. The surface geometry was then imported into ABAQUS/Standard 2019 where it was converted from a shell (.stl) to a solid (.sat) using the “create a geometry from mesh” software plugin. The dimensions of all the parts of the model are reported in Supplementary Data.

All biological tissues were modelled as poroelastic materials with properties given in Table 1. The fixator, the nails and the scaffolds were considered linear elastic isotropic and modelled identical in both scaffold designs. Scaffolds were assumed to be made of Polycaprolactone (PCL), a material highly investigated for scaffold applications (Dwivedi et al., 2020), while the fixator and the nails were assumed to be made of Polyether-ether-ketone (PEEK) and titanium, respectively, following a previous pre-clinical experimental study (Schwarz et al., 2013). Therefore, the material properties of Polyether-ether-ketone: PEEK (E = 3,800 MPa, v = 0.3), titanium: Ti (E = 111,000 MPa, v = 0.3), and Polycaprolactone: PCL (E = 350 MPa, v = 0.33) were assigned to the fixator, the nails and the scaffolds, respectively.

**TABLE 1:** Tissue material properties [adapted from (Checa et al., 2011)].

Material properties	Granulation tissue	Fibrous tissue	Cartilage	Immature bone	Mature bone	Cortical bone	Bone marrow
Young’s modulus (MPa)	0.2	2	10	1,000	5,000	5,000	2
Permeability (10^−14^ s m^4^/N)	1	1	0.5	10	37	0.001	1
Poisson’s ratio	0.167	0.167	0.3	0.3	0.3	0.3	0.167
Bulk modulus grain (MPa)	2,300	2,300	3,700	13,940	13,940	13,920	2,300
Bulk modulus fluid (MPa)	2,300	2,300	2,300	2,300	2,300	3,200	2,300

Mechanical loading conditions aimed to simulate the peak load under normal walking conditions. An axial compressive load of 14.7 N [corresponding to 6 body weight (BW)] was applied at the proximal bone side (Wehner et al., 2010) (Figure 1). In addition, two tangential forces of 1.8 N were applied on the proximal bone surface in the antero/posterior and in the medial/lateral directions thereby inducing bending loads (corresponding to 10.7 BW mm of moment at the femoral mid-shaft) (Wehner et al., 2010) (Figure 1). The distal part was fully constrained. A pore pressure boundary condition was constrained to be zero on the external surface of the callus domain.

The model was meshed using three-dimensional quadratic tetrahedral elements (C3D10 MP) with an average mesh size of 0.50 mm for the whole model except for the scaffold and the callus region, which had an average mesh size of 0.20 mm.

Scaffolds were assumed to experience bulk degradation where scaffold’s volume is preserved, as reported for PCL (Pitt et al., 1981). To model this, the mechanical properties of the polymer were assumed to be linearly related to its molecular weight (Adachi et al., 2006). Scaffold degradation was then simulated using the following equation:

$$E=E_{0}e^{-kt}$$

With *E* the updated Young’s modulus, $E_{0}$ the initial Young’s modulus, *k* the degradation rate per day and *t* for time. A degradation rate of 0.003 day^−1^ was assumed as reported for PCL material in an *in vivo* setting (Pitt et al., 1981).

#### 2.1.2 Agent-based computer model to investigate cellular activity within the scaffold pores

An agent-based computer model was implemented using C++, where the space occupied by the scaffold and the regenerating tissue region (scaffold pores) was discretized into a 3D grid (spacing 10 µm) in which each of the positions within the scaffold pores represents a potential space a cell could occupy. This means that the space occupied by each element in the FE model would contain a number of agents in the agent based model. Since each agent size is 10 × 10 × 10 um^3^, the FE callus dimensions (x = 6mm, y = 6 mm and z = 7.2 mm) were translated into the ABM to 600 agents in the x direction, 600 agents in the y direction and 720 agents in the z direction; resulting in a total of 259200000 agents in the 3D grid. The following cell phenotypes were included: mesenchymal cells (MSC), fibroblasts, chondrocytes, immature osteoblasts and mature osteoblasts. The model simulates cellular processes including migration, proliferation, differentiation and apoptosis. Cell differentiation, proliferation and apoptosis are regulated by a mechanical stimulus based on octahedral shear strain and fluid flow extracted from the FE model (Checa et al., 2011) (Table 2). Cell differentiation was modelled to occur only on top of existing surfaces (scaffold or newly formed tissues), simulating surface-guided bone regeneration within scaffolds (Perier-Metz et al., 2020). In addition, different cellular processes are modelled to occur at different rates (Table 3).

**TABLE 2:** Mechano-regulation algorithms for progenitor cell differentiation [adapted from (Checa et al., 2011)].

Stimulus: $S=\frac{\gamma }{a}+\frac{\nu }{b}$	Bone resorption	Mature osteoblast	Immature osteoblast	Chondrocyte	Fibroblast
γ : shear strain, ν : fluid velocity					
a = 0.0375^a^, b = 0.003 mm/s^a^					
Thresholds^a^ ^,^ ^b^	S≤0.01	0.01<S≤2.53	2.53<S≤3	3<S≤5	S>5

a (Huiskes et al., 1997).

b (Lacroix and Prendergast, 2002).

**TABLE 3:** Cell activity rates [adapted from (Checa et al., 2011)].

Cell type	Proliferation rate (/day)	Apoptosis rate (/day)	Differentiation rate (/day)	Migration speed (µm/h)
MSC	0.60	0.05	0.30	30
Fibroblasts	0.55	0.05	—	30
Chondrocytes	0.20	0.10	—	—
Osteoblasts	0.30	0.16	—	—

To simulate the invasion of MSCs from the marrow cavity and periosteum, 30% of the agent-based positions along the periosteum and marrow cavity were initially seeded with MSCs (Checa et al., 2011). In addition, following Perier-Metz et al. (2020), the scaffold pores were assumed to be filled with bone graft (Finkemeier, 2002). The bone grafting effect on the bone regeneration process was modelled by limiting progenitor cell migration and proliferation to the regions containing graft (scaffold pores) after a latency period (14 days) (Perier-Metz et al., 2020).

Cells were simulated to produce the corresponding extracellular matrix (osteoblasts: bone, chondrocytes: cartilage and fibroblasts: fibrous tissue), so that each cell position was assumed to account for corresponding extracellular matrix deposition. Extracellular matrix deposition is modelled as cell differentiation of MSCs into another cell type and a change in tissue material properties in the FE model; which were updated iteratively (1 iteration = 1 day). All differentiated cells inside each single element contributed to the element material properties, following a rule of mixtures (Lacroix and Prendergast, 2002). In addition, the contribution of each differentiated cell to the specific element material properties were averaged over the last ten iterations to account for the delay in actual ECM deposition (Lacroix and Prendergast, 2002). Therefore, the agent-based and the FE models interacted through the level of mechanical signals (from the FEM to the ABM) and the corresponding changes in tissue material properties (from the ABM to the FEM).

#### 2.1.3 Scaffold design evaluations

The effect of each scaffold parameter was assessed following common experimental setups in which all variable factors (eg. material type, porosity, pore size…) in an experimental group (eg. gyroid scaffold) and a comparison control group (eg. strut-like scaffold) are kept the same except for one variable factor (eg. architecture) that differs between the two groups. Accordingly, several scaffold design effects were investigated (Table 4):

• Scaffold architecture effect: To examine the influence of the scaffold architecture on bone tissue regeneration, two scaffolds were modelled by assuming the same scaffold porosity and material parameters and varying the scaffold architecture. A model with a gyroid architecture was compared to a model with a strut-like architecture (Figure 1).
• Scaffold degradation effect: Since the degradation rate of PCL material is very slow, the effect of degradation on the bone regeneration process was expected to be minimal. Thereby, to investigate the effect of scaffold degradation on the regeneration process, the influence of a highly degradable scaffold (k = 0.03 per day) on bone regeneration was compared to the effect of a non-degradable scaffold, both with the same gyroid design.
• Porosity effect: To examine the influence of scaffold porosity on the bone regeneration process, both gyroid and strut-like scaffolds were modelled with a lower porosity of ∼69% and compared with the 79% porous scaffolds. The gyroid scaffold with 69% porosity had a pore size of 0.5 mm and a surface area to volume ratio of 2.43, while the strut-like scaffold had a pore size of 0.49 mm and a surface area to volume ratio of 4.3.

**TABLE 4:** Summary of the bone healing simulations and scaffold characteristics.

Name	Architecture	Porosity (%)	Degradation	Pore size	Number of nodes	Number of elements
Gyroid scaffold	Gyroid	79	Normal	0.54	417,684	301,748
Strut-like scaffold	Strut-like	79	Normal	0.56	372,137	267,151
Highly degradable scaffold	Gyroid	79	Fast	0.56	417,684	301,748
Non-degradable scaffold	Gyroid	79	—	0.56	417,684	301,748
Gyroid scaffold with lower porosity	Gyroid	69	Normal	0.5	443,353	320,911
Strut-like scaffold with lower porosity	Strut-like	69	Normal	0.49	368,107	263,877

### 2.2 Output data analysis

#### 2.2.1 Overall scaffold stiffness computation

A virtual compression test was performed to compute the overall stiffness of the scaffolds and their changes over the healing process. The compression test was done by applying a vertical force (F = 15 N) at the top surface of the scaffolds and outputting the resultant average vertical displacement.

#### 2.2.2 Tissue regeneration

Tissue distribution in a longitudinal section through the middle of the scaffold (Figure 1A) was evaluated and represented in a similar manner to histological sections. Tissue patterning was evaluated at 4 time points: 0-, 4-, 8-, and 12-weeks post-surgery. Moreover, the relative area occupied by the different tissue types within the mid-section in the callus and the total volume of the different tissue types within the scaffold pores were quantified daily until 12 weeks.

Cellular activities happening within the scaffold pores were quantified by measuring the total number of cells that migrated (regardless of distance travelled), proliferated, differentiated to other cell types and died daily until 12 weeks. All MSCs and fibroblasts are potential migrating cells but only those that manage to change position within one iteration are considered migrating cells. The cell were assigned a migrating speed of 30 microns/hr which is equivalent to 720 microns/day. Also, the average speed of the cells (MSCs and fibroblasts) was quantified by dividing the average distance travelled by the cells by the number of cells that travelled per day.

#### 2.2.3 Mechanical stimulus

In order to investigate the changes in the mechanical conditions within the scaffold pores during tissue regeneration, the mechanical stimulus (based on octahedral shear strain and fluid flow) distribution was computed 0-, 4-, 8-, and 12-weeks post-surgery, for each of the scaffold designs investigated.

## 3 Results

### 3.1 Predicted tissue formation over the course of healing

#### 3.1.1 Gyroid vs. strut-like scaffolds

**FIGURE 2:**
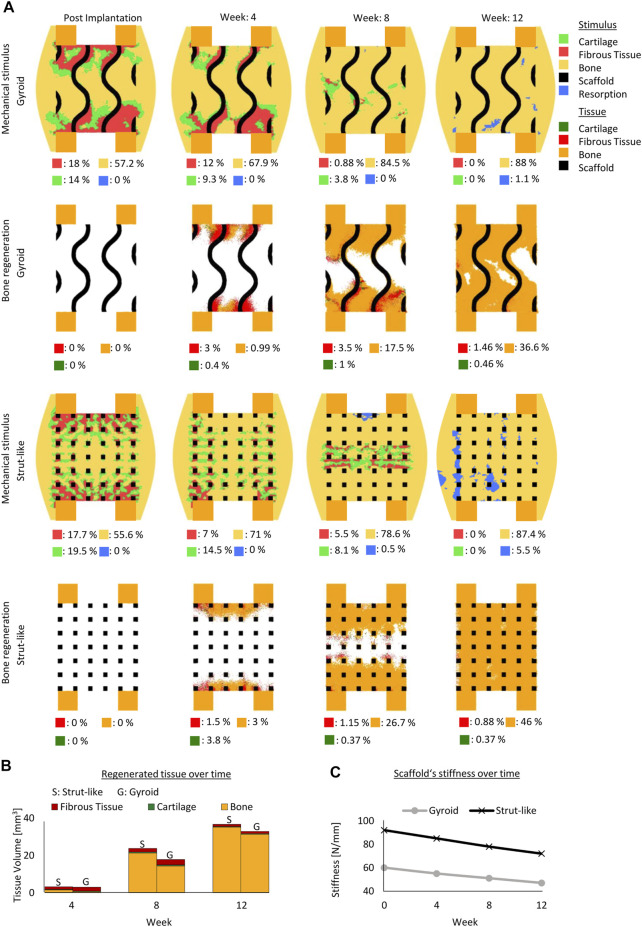
Predicted bone, cartilage and fibrous tissue within the scaffold pores at 4, 8 and 12 weeks of healing for scaffolds with strut-like and gyroid architecture with 79% porosity. **(A)**: Longitudinal cross sections of the callus from Figure 1A with quantified tissue regeneration in the 2D section, mechanical stimulus distribution over time using a strut-like scaffold, predicted tissue distribution over time using a strut-like scaffold, mechanical stimulus distribution over time using a gyroid scaffold and predicted tissue distribution over time using a gyroid scaffold. **(B)**: Total tissue volume predicted within the scaffold pores at different stages of healing. **(C)**: Mechanical stiffness of scaffolds computed from compression tests at different stages of healing.

Initial mechanical stimuli distribution within the gyroid and strut-like scaffolds were different, despite being under the same external mechanical load (Figure 2A). Initially, in both scaffold designs, most of the tissue volume was under mechanical stimuli beneficial for bone formation; however, the distribution across the defect highly varied (Figure 2A). Higher mechanical signals beneficial for cartilage and fibrous tissue formation were detected at the contact region between the cortical bone and scaffold in both scaffold designs (Figure 2A).

These differences in the mechanical behavior of the scaffolds and the induced strains within the scaffold pores resulted in a divergence of the bone healing progression. In the strut-like scaffold, bone tissue started to form within the scaffold pores, simultaneously starting from the top and bottom surfaces and slowly progressing towards the core region by intramembranous ossification. Only small regions of fibrocartilage layers that would surround the scaffold walls were predicted. However, in the gyroid scaffolds, large fibrous tissue volumes were predicted to initially form surrounding the highly curved scaffold surfaces, with bone formation predicted to occur in regions far from those surfaces (Figure 2A). For the gyroid scaffolds, fibrous tissue was predicted to be slowly replaced by bone over the course of healing.

The healing outcome was also considerably different between the two scaffolds. After 12 weeks, bony bridging was observed in the strut-like scaffold, which had already entered the remodeling phase, whereas void regions were observed within the scaffold core of the gyroid scaffold. Overall, quantitatively more bone formation was predicted within the strut-like scaffold compared with the gyroid scaffold (Figures 2A.

The *in silico* longitudinal compression test of the scaffolds showed that during healing the strut-like scaffold provided a higher total stiffness compared the gyroid scaffold (Figure 2C).

#### 3.1.2 Degradation effect

**FIGURE 3:**
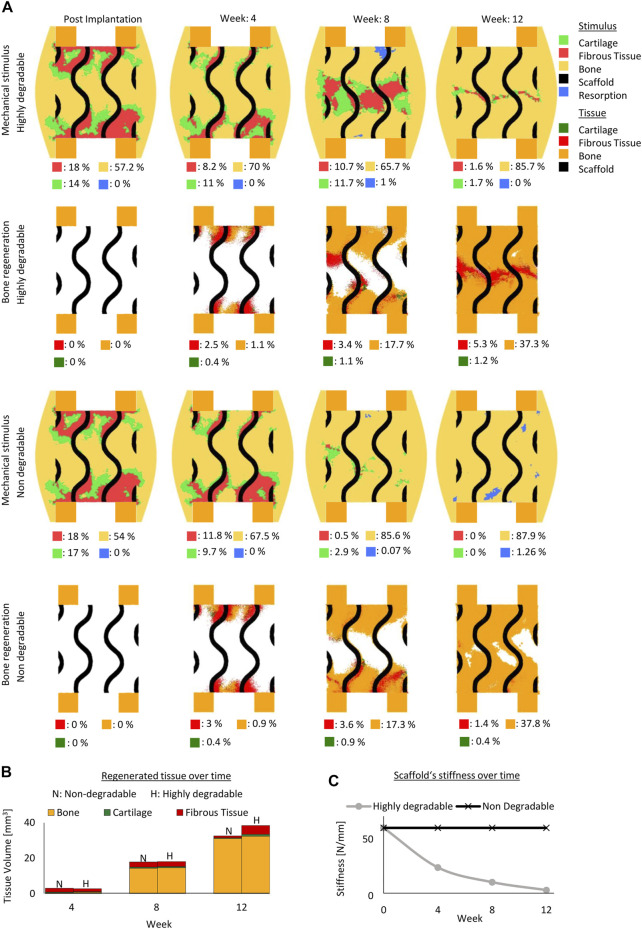
Predicted bone, cartilage and fibrous tissue within the scaffold pores at 4, 8 and 12 weeks of healing for scaffolds with non- and highly degradable scaffolds with 79% porosity. **(A)**: Longitudinal cross sections of the callus from Figure 1A with quantified tissue regeneration in the 2D section, mechanical stimulus distribution over time using a non-degradable scaffold, predicted tissue distribution over time using a non-degradable scaffold, mechanical stimulus distribution over time using a highly degradable and predicted tissue distribution over time using a highly degradable. **(B)**: Total tissue volume predicted within the scaffold pores at different stages of healing. **(C)**: Mechanical stiffness of scaffolds computed from compression tests at different stages of healing.

Initially, the mechanical stimuli within the fast degradable and non-degradable gyroid scaffolds were comparable (Figure 3A). With the loss of the mechanical stiffness of a fast degradable scaffold over time, a noticeable difference in the mechanical stimuli distribution between a fast degradable and a non-degradable scaffold was observed (Figure 3A). The non-degradable scaffold led to more regions beneficial for bone formation, whereas the fast degradable scaffold resulted in more regions beneficial for fibrous tissue formation (Figure 3A). Across the early healing stages, the amount of predicted regenerated tissue within both scaffold configurations differed slightly (Figures 3A. However, at late stages of healing, the total amount of tissue regenerated highly varied (Figures 3A. After 12 weeks, the highly degradable scaffold yielded only slightly more bone tissue compared to the non-degradable scaffold, however it resulted in considerably more fibrous tissue. While a layer of fibrous tissue was observed at the end of the regeneration process in the highly degradable scaffold, regions with remaining voids were found in a non-degradable scaffold (Figures 3A.

In addition, even though the fast degradable scaffold was undergoing an exponential decay in its mechanical stiffness reaching 5 N/mm at the end of the regeneration process (initially 60N/mm) (Figure 3C), the formation of tissue at the early stages of healing maintained a stable mechanical environment beneficial for new bone formation within the defect region.

#### 3.1.3 Porosity effect

**FIGURE 4:**
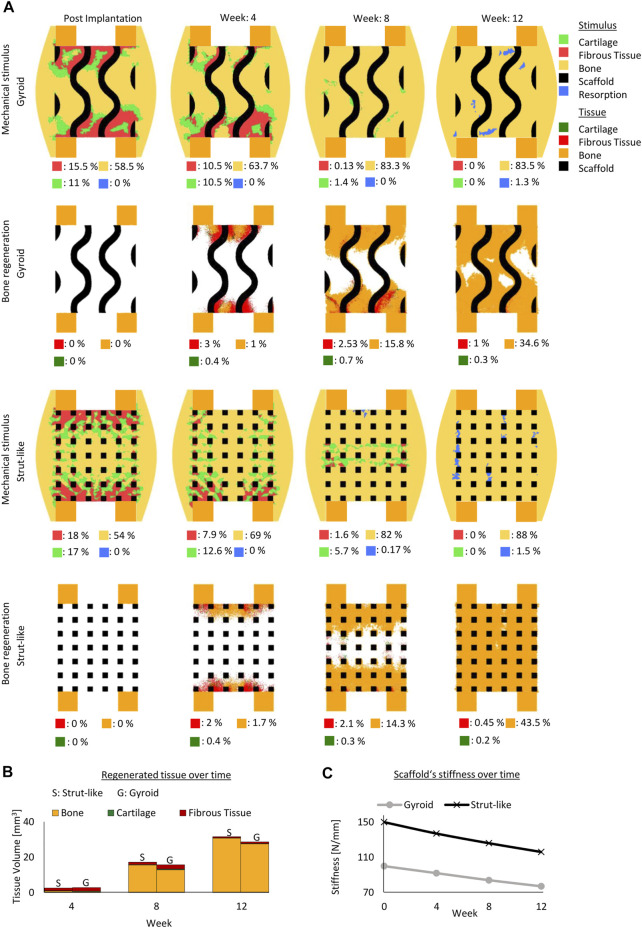
Predicted bone, cartilage and fibrous tissue within the scaffold pores at 4, 8 and 12 weeks of healing for scaffolds with strut-like and gyroid architecture with 69% porosity. **(A)**: Longitudinal cross sections of the callus from Figure 1A with quantified tissue regeneration in the 2D section, mechanical stimulus distribution over time using a strut-like scaffold, predicted tissue distribution over time using a strut-like scaffold, mechanical stimulus distribution over time using gyroid scaffold and predicted tissue distribution over time using a gyroid scaffold. **(B)**: Total tissue volume predicted within the scaffold pores at different stages of healing. **(C)**: Mechanical stiffness of scaffolds computed from compression tests at different stages of healing.

In the gyroid design, a scaffold with 69% porosity resulted in initial mechanical stimuli similar to a scaffold with 79% porosity (Figures 2A, 4A). With both porosities, most of the predicted tissue forming during the regeneration process was predicted to be bone. With the higher porosity scaffold more bone was formed compared to the one with the lower porosity scaffold (Figures 2A, 4A) Although, the predicted mechanical stimulus within both scaffold porosities continued to be similar during the regeneration process (Figures 2A, 4A), the model with lower porosity yielded less bone and fibrocartilage tissue due to slower tissue formation penetrating the scaffold and resulting in overall less tissue formation (Figures 2A, 4A. The mechanical stimuli in both scaffolds predicted mostly bone formation with distinct certain regions in the resorption zone at the end of the regeneration process (Figures 2A, 4A).

Similarly, in the strut-like designs, the mechanical stimuli were comparable for both scaffold porosities settings across the regeneration process (Figures 2A, 4A).While bone bridging was observed within the higher porosity scaffold at the end of the regeneration process, void regions at the center of the defect were observed within the lower porosity scaffold (Figures 2A, 4A).

In both scaffold design, a 10% reduction in scaffold porosity led to an increase of 40% in the overall scaffold stiffness (Figure 4C).

### 3.2 Analysis of cellular activity

The cellular activities including MSC migration, osteoblast proliferation and differentiation, and fibroblast differentiation were quantified per day in different scaffold designs.

#### 3.2.1 Mesenchymal cells migration

**FIGURE 5:**
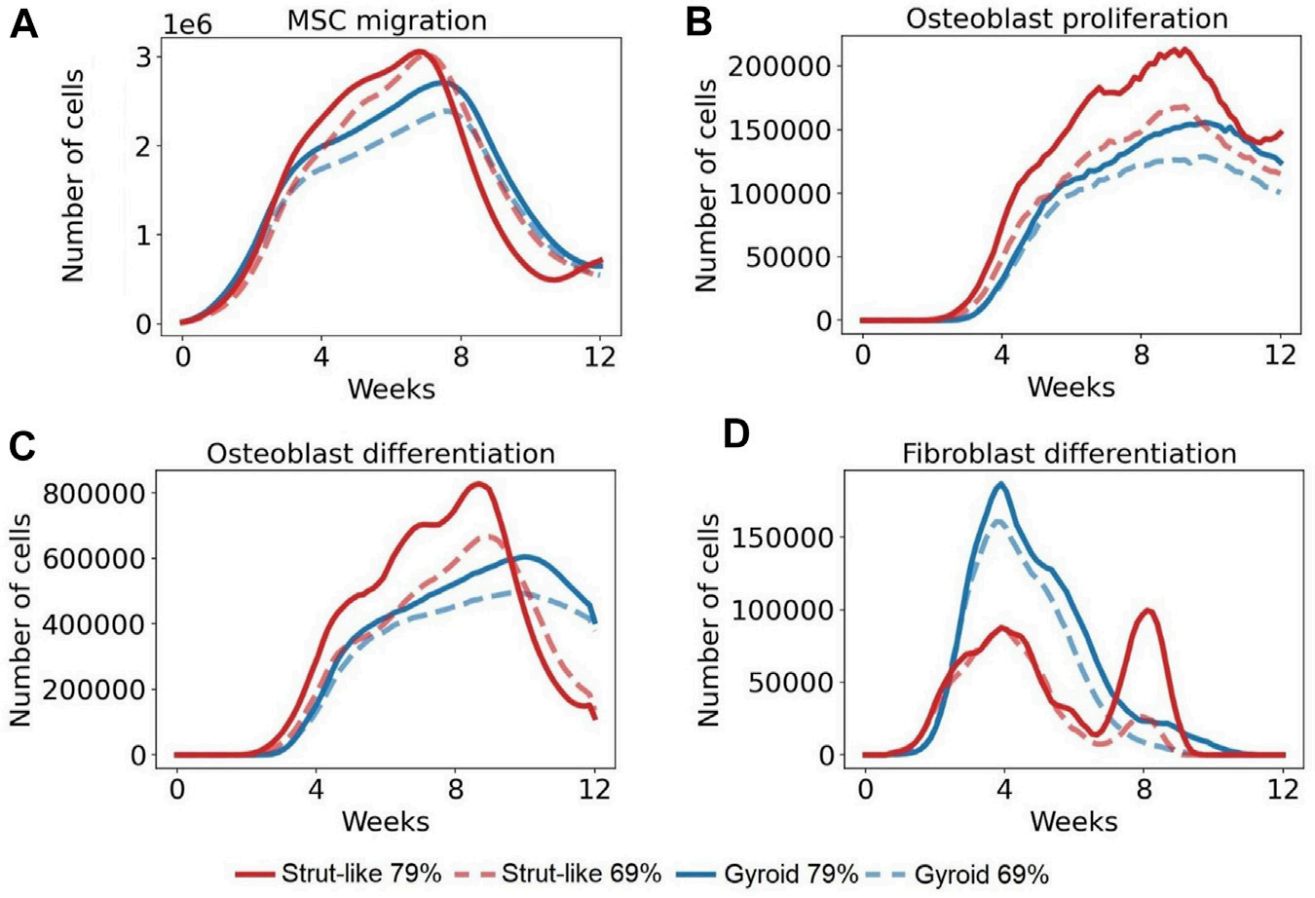
The number of the total cells per day across time in several cellular activities. **(A)**: Total number of MSCs that migrated per day in 12 weeks **(B)**: Total number of osteoblasts that proliferated per day in 12 weeks. **(C)**: Total number of MSCs that differentiated into osteoblasts per day in 12 weeks **(D)**: Total number MSCs that differentiated into fibroblasts per day in 12 weeks.

Initially, the number of predicted MSCs migrating was similar in all scaffold configurations, however, higher MSC migration was predicted around the 3rd week in strut-like scaffolds. The cell migration continued to increase over the following weeks, with a higher increase within the strut-like compared with the gyroid scaffolds (Figure 5A). At the 8th–9th week, a decrease in the cell migration was predicted until the end of the regeneration process, for all scaffold designs. In addition to scaffold architecture, scaffold porosity showed a strong effect on MSC migration. Scaffolds with higher porosity showed increased migration, especially during the middle phase of the regeneration process (Figure 5).

#### 3.2.2 Osteoblasts proliferation

Initially, the number of predicted osteoblasts proliferating was similar in all scaffold configurations. Whereas strut-like and gyroid scaffolds had comparable osteoblastic proliferation behaviour, over the course of the healing, higher osteoblastic proliferation was observed within the strut-like scaffold. In both scaffold architectures, the number of osteoblasts proliferating increased until the 9–10th week, from this time point, a decrease in the number of osteoblasts proliferation was observed until the end of the regeneration process. Similar to MSC migration, increase in the scaffold porosity lead to increase osteoblast proliferation in both scaffold architectures, however the effect was larger in the strut-like design (Figure 5B).

#### 3.2.3 Osteoblasts differentiation

Initially, the number of MSCs differentiated into osteoblasts was similar in all scaffold configurations, however at the later stages of regeneration considerably more osteoblastic differentiation was predicted to occur within the strut-like scaffolds. In strut-like scaffolds, a considerable increase in osteoblast differentiation was predicted around the 8th–9th week, where a much lower peak was predicted around the 10th week within the gyroid scaffolds. Similar to MSC migration and osteoblast proliferation, increase scaffold porosity led to increase osteoblast differentiation in both scaffold architectures, however the effect was larger in the strut-like design (Figure 5C).

#### 3.2.4 Fibroblasts differentiation

The number of MSCs differentiated into fibroblasts increased during the initial healing period in all scaffolds configurations (Figure 5D), however, higher fibroblast differentiation was predicted around the 4th week in gyroid scaffolds. From this time point, fibroblasts differentiation decreased until the end of the regeneration process. However, the strut-like scaffold showed a different trend, where at around the 6th–8th week had a second increase, followed by a decrease until the end of the regeneration process. Furthermore, lower fibroblast differentiation was predicted within the scaffolds with lower porosity, with higher difference within the strut-like design (Figure 5D).

Quantification of other cellular activities over the course of regeneration for the different scaffold designs is provided in Supplementary Data. The cellular activities include fibroblasts, chondrocytes and MSCs proliferation, fibroblasts, chondrocytes and osteoblasts apoptosis, fibroblasts and MSCs average speed during migration, MSCs differentiation into chondrocytes and number of migrated fibroblasts.

Cumulative number of proliferated, differentiated and dead cells is reported in Supplementary Data.

## 4 Discussion

In the recent years, gyroid architectures have gained a lot of interest in tissue engineering for their high potential to be used as scaffolds in the treatment of large bone defects (Castro et al., 2019b; Yang et al., 2019; Jin et al., 2020). However, so far there has been no study to investigate whether they present advantages over traditional scaffolds for their bone regeneration potential. In this study, we use the power of computer modelling approaches to investigate whether a gyroid as a scaffold architecture presents an advantage, in terms of healing outcome, over a traditional scaffold with a strut-like architecture. Contrary to our initial hypothesis, our results showed reduced predicted healing outcome in gyroid compared to strut-like scaffolds.

In this study, we adapted a previously described bone regeneration computer model, which was able to explain experimentally observed scaffold-supported bone regeneration for two different scaffold designs in two independent pre-clinical studies of bone regeneration within large bone defects (Perier-Metz et al., 2020; Perier-Metz et al., 2022), to predict bone regeneration within a large bone defect supported with a gyroid scaffold. The model was previously able to explain bone regeneration within a titanium honeycomb scaffold and a PCL strut-like scaffold, where surface-scaffold guidance was identified as an important mechanism during scaffold supported regeneration (Pobloth et al., 2018; Perier-Metz et al., 2020; Perier-Metz et al., 2022; Pobloth et al., 2018). Other mechanoregulation theories have been previously proposed to explain the mechanobiological regulation of bone regeneration (Carter et al., 1998; Claes and Heigele, 1999; Prendergast et al., 1997). Mechanical stimuli based on hydrostatic stress and tensile strain, mechanical strains and hydrostatic pressure, and octahedral strain and fluid velocity have all been proposed as potential regulators. Computer modeling approaches have been used to test those theories and their potential to predict *in vivo* tissue regeneration, where it has been shown that most of those theories result in comparable tissue regeneration prediction, which in general agrees with experimental observations (Geris et al., 2003; Isaksson et al., 2006b; Postigo et al., 2014). In this study, we quantified regions under tension and compression strains and found that tensile strains were higher at the scaffold bone interface, while compressive strains were higher between the scaffold walls (Supplementary Data).

Unfortunately, experimental studies on scaffold-supported bone regeneration within gyroid scaffolds are very limited. To the authors knowledge, the only *in vivo* study that compared the bone healing potential of a gyroid scaffold to a strut-like scaffold is the one by Van hede et al. (2022), where a skull defect was used as defect model. Since skull bone has different developmental and regenerative mechanisms than long bones (Lim et al., 2013), those *in vivo* observations cannot be compared to the computer model predictions presented here. *In vitro* experiments of cellular function within gyroid scaffolds have been mainly performed to test different biomaterials or 3D printing techniques, where in general it has been reported that cells are able to penetrate and proliferate within gyroid scaffolds (Melchels et al., 2010a; Tsai et al., 2015; Ataee et al., 2019; Diez-Escudero et al., 2020; Spece et al., 2020; Chen et al., 2021; Noroozi et al., 2022). Spece et al. (2020) compared cellular activity within gyroid and strut-like scaffolds in terms of ALP activity, where they showed higher ALP activity in strut-like scaffolds. These findings agree with the higher osteoblast activity in strut-like scaffolds predicted in this study.

In this study, gyroid and strut-like scaffolds of the same porosity and almost the same pore size were compared in terms of predicted bone regeneration. In the gyroid scaffolds, the pore size does not describe the distance between surfaces, in fact in the scaffolds investigated here the distance between the surfaces for the gyroid scaffolds were approximately twice the pore size. This has a major impact not only in reducing the overall mechanical properties but also in the surface area to volume ratio and the cellular behaviour. Reduced surface area to volume ratio was determined in the gyroid compared with the strut-like scaffolds for a given porosity. In this study, we predicted reduced cell proliferation in gyroid compared to strut-like scaffolds, where strut-like scaffolds had a larger surface area to volume ratio. This is in agreement with experimental studies that reported faster cell growth for shorter distance between scaffold inner surfaces (Van Bael et al., 2012; Liu et al., 2020).

Since various polymeric materials used as scaffold materials experience degradation (Pitt and Zhong-wei, 1987), degradation due to hydrolysis undergoing exponential decay was implemented in the model (Adachi et al., 2006). Given that PCL degrades very slowly (degradation rate constant: 0.003 per day) (Pitt et al., 1981), the effect of its degradation on tissue formation was negligible. The effect of scaffold degradation on tissue regeneration was marked by comparing a highly degradable (0.03 per day) to a non-degradable scaffold. Although, this degradation rate is unrealistic for PCL, the scaffold material properties were kept constant to isolate the effect of degradation. The highly degradable scaffold resulted in more soft tissue and overall tissue formation compared with the non-degradable scaffold. Scaffold degradation was predicted to affect bone regeneration by decreasing the scaffold’s mechanical stiffness allowing for higher deformations promoting soft tissue formation within the defect region. Our results showed that a highly degradable scaffold led to more bone regeneration, which is in agreement with experiments done on polymeric scaffolds where they found that at 3 months, the maximum amount of bone was observed in the scaffold with the highest degradability (Huang et al., 2019).

In this study, scaffolds with two different porosities (69% and 79%), both for gyroid and strut-like designs, were investigated. We found that while there was a substantial difference in the overall mechanical properties of the scaffolds, in agreement with experimental studies (Baino and Fiume, 2019), the mechanical environment within the scaffold pores for different porosities was similar. However, due to larger pores sizes in the scaffolds with higher porosities, more penetration of the cells was predicted, leading to more bone regeneration. This is also in agreement with experimental findings (Lee et al., 2019). Pobloth et al. (2018) showed that scaffolds with lower porosity but with the same architecture yield less bone and slower healing dynamics. Furthermore, we found a lower influence of scaffold porosity on tissue regeneration in the gyroid compared to the strut-like scaffold which could be explained by the smaller changes in surface area to volume ratio with changes in porosity in those scaffolds.

In addition, our results show reduced mechanical stiffness in the gyroid compared with the strut-like scaffolds, in agreement with numerical studies (Ali and Sen, 2017). With the same porosity, the strut-like scaffold was stiffer than the gyroid scaffold; however, the former scaffold showed considerably more bone formation than the latter. Previous *in vivo* studies have shown that softer scaffolds can enhance bone regeneration (Pobloth et al., 2018). This study shows that the scaffold architecture can have a higher impact on bone formation than overall scaffold stiffness.

Our study presents some limitations. First, in contrast to studies suggesting higher surface area to volume ratio for the gyriod scaffold (Lu et al., 2020; du Plessis et al., 2018), in this study, the gyroid scaffold had lower surface area to volume ratio. This is because in our models, to keep the porosity and pore size similar in both the gyroid and strut-like scaffolds, the number of scaffold unit cells was different for each scaffold design which led to lower surface area to volume ratio in the gyroid scaffold as compared to the strut-like scaffold. Previous studies have shown that by fixing the porosity and varying the unit cells of the scaffold, the scaffold mechanical properties are altered (Jian et al., 2015) and consequently, the bone regeneration (Pobloth et al., 2018). We performed additional simulations to investigate the effect of the number of scaffold unit cells. For a given porosity, we found that increasing the number of unit cells increases the surface area to volume ratio and enhances bone regeneration, both in gyroid and strut-like configurations (Supplementary Data). However, although the gyroid scaffold presented higher surface to volume ratio (gyroid: 3.32 and strut-like: 2.58), the strut-like scaffold still resulted in more bone formation compared to the gyroid scaffold for comparable number of unit cells (Supplementary Data). A further limitation relates to the size of the parametric analysis performed. In this study, each scaffold design parameter was studied across two variations only (architecture: gyroid and strut-like; porosity: 79 % and 69%; degradation: degradable and non-degradable). Although, the number of models were sufficient to test this study’s main hypothesis on whether gyroid scaffold outperform strut-like scaffolds, further studies with higher ranges of scaffold design parameters could give a better understanding on the interplay between the scaffolds design parameters and identify which scaffold design parameter has the highest impact on the bone regeneration. Finally, among the limitations of the study is using the same degradation rate in all scaffold configurations. Wu and Ding (2005) showed that increasing the pore size or decreasing the porosity lead to an increase in the rate of degradation in PLGA materials. However, since the material used here is PCL, which has a very slow degrading rate, this effect does not influence the results.

In conclusion, here we used a computer model of scaffold-supported bone regeneration, that was previously able to explain bone regeneration within two different scaffold designs in two experimental setups, to predict, for the first time, the bone regeneration potential of PCL gyroid scaffolds in long bone large defects. Computer model predictions suggest slower healing dynamics and reduced bone tissue formation in gyroid compared with strut-like scaffolds. Future studies should focus on the experimental validation of these findings so that they can be used for the optimization of scaffold design to support bone regeneration in long bone large defects.

## Data Availability

The original contributions presented in the study are included in the article/Supplementary Material; further inquiries can be directed to the corresponding author.
