# Supplementary material for: PCL strut-like scaffolds appear superior to gyroid in terms of bone regeneration within a long bone large defect: An in silico study
Source: Front Bioeng Biotechnol. 2022 Sep 23;10:995266. doi: 10.3389/fbioe.2022.995266 (PMC9540363; doi:10.3389/fbioe.2022.995266)
Supplement: Supplementary file 1 [file DataSheet1.docx]

Supplementary Material

**Supplementary Figure 1.** Experimental setup dimensions.


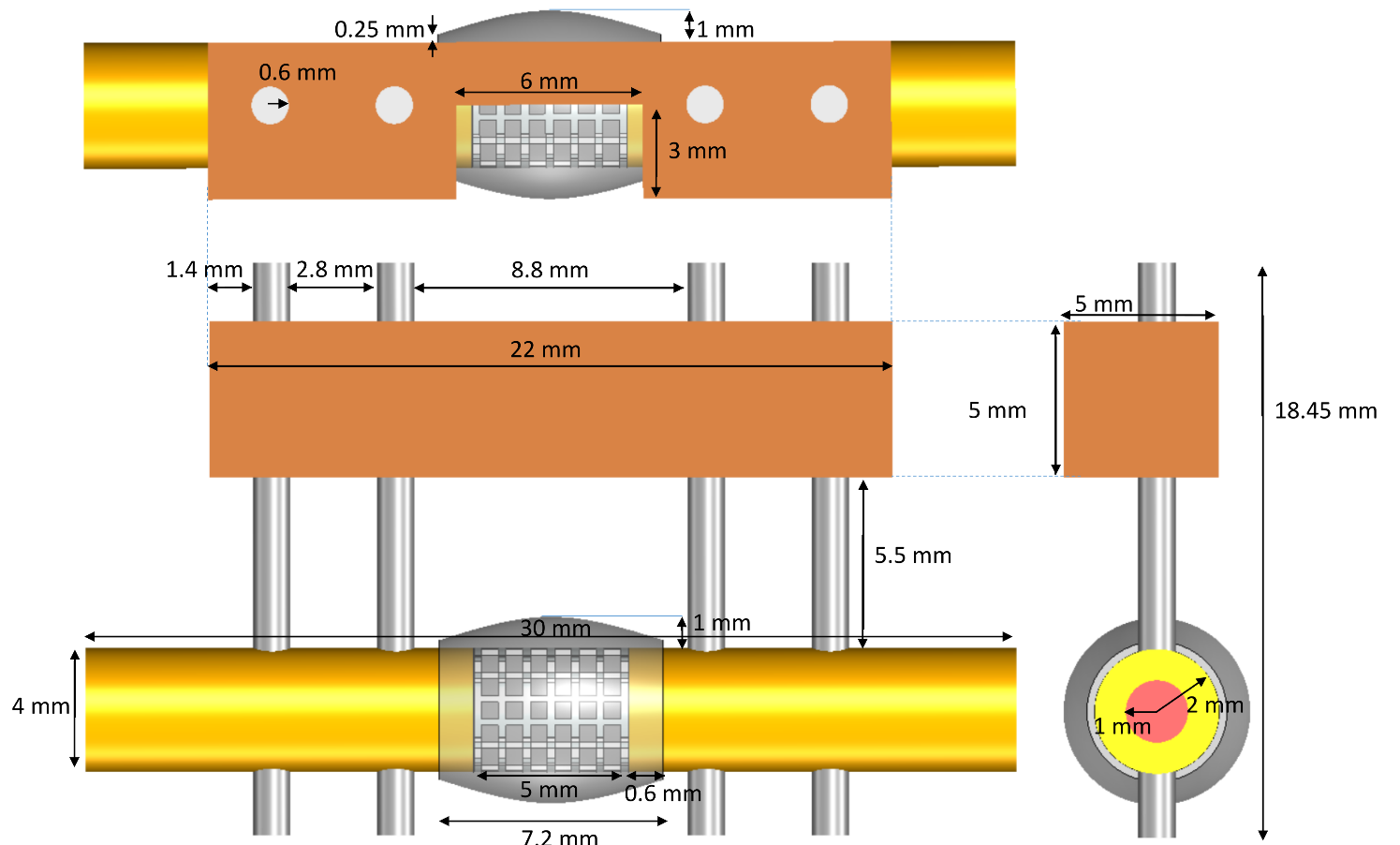


**Supplementary Figure 2.** Several cellular activities quantified per day across 12 weeks. (A): Total number of fibroblasts that proliferated per day in 12 weeks. (B): Total number of chondrocytes that proliferated per day in 12 weeks. (C): Total number of osteoblasts that proliferated migrated per day in 12 weeks. (D): Total number of fibroblasts that died per day in 12 weeks. (E): Total number of chondrocytes that died per day in 12 weeks. (F): Total number of osteoblasts that died per day in 12 weeks. (G): Average speed of the total number of migrated fibroblasts per day in 12 weeks. (H): Average speed of the total number of migrated MSCs per day in 12 weeks. (I): Total number of chondrocytes that differentiated per day in 12 weeks. (J): Total number of fibroblasts that migrated per day in 12 weeks.


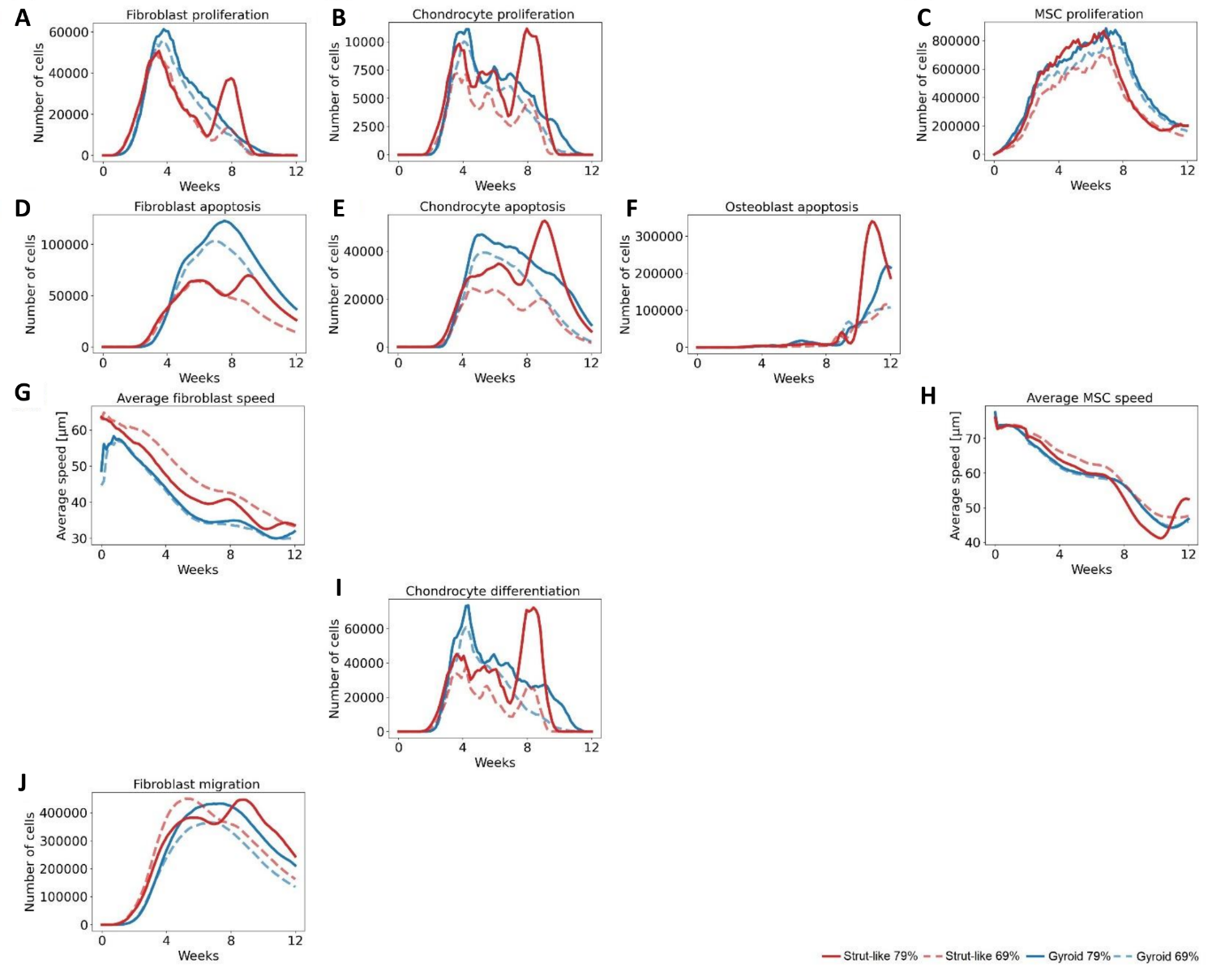


**Supplementary Figure 3.** Several cellular activities quantified cumulatively per day across 12 weeks. (A): Total number of fibroblasts that proliferated per day in 12 weeks. (B): Total number of chondrocytes that proliferated per day in 12 weeks. (C): Total number of osteoblasts that proliferated migrated per day in 12 weeks. (D): Total number of MSCs that proliferated migrated per day in 12 weeks. (E): Total number of fibroblasts that died per day in 12 weeks. (F): Total number of chondrocytes that died per day in 12 weeks. (G): Total number of osteoblasts that died per day in 12 weeks. (H): Total number of fibroblasts that differentiated per day in 12 weeks. (I): Total number of chondrocytes that differentiated per day in 12 weeks. (J): Total number of osteoblasts that differentiated per day in 12 weeks.


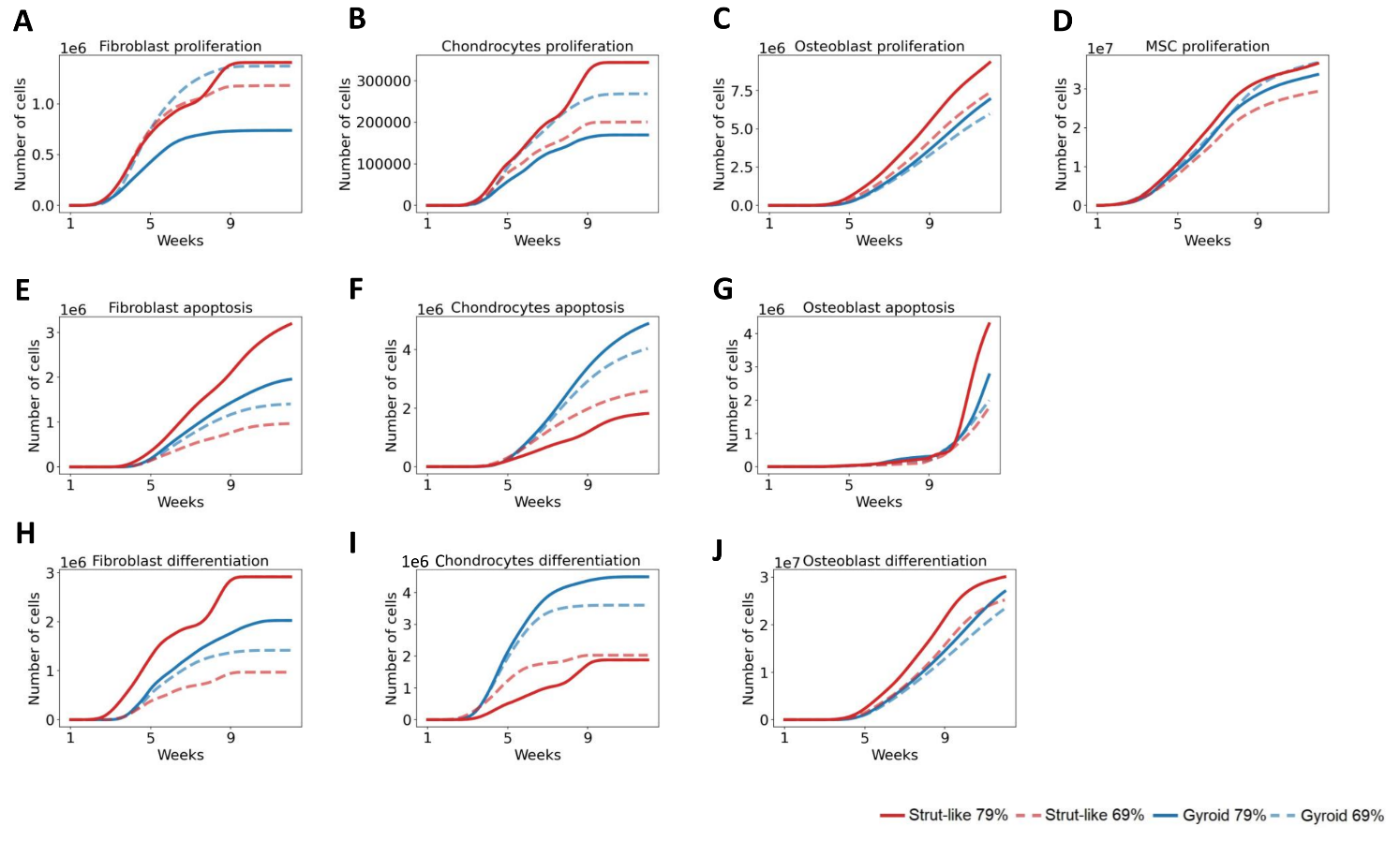


**Supplementary Figure 4.** Predicted bone, cartilage and fibrous tissue within the scaffold pores at 4, 8 and 12 weeks of healing for scaffolds with strut-like designed with 3 - 4 unit cells and gyroid architecture designed with 2 - 3 unit cells, both with 79% porosity. (A): Longitudinal cross sections of the callus from Figure 1 (A) with quantified tissue regeneration in the 2D section, Mechanical stimulus distribution over time using a strut-like scaffold, Predicted tissue distribution over time using a strut-like scaffold, Mechanical stimulus distribution over time using gyroid scaffold and Predicted tissue distribution over time using a gyroid scaffold. (B): Total tissue volume predicted within the scaffold pores at different stages of healing. (C): Mechanical stiffness of scaffolds computed from compression tests at different stages of healing.


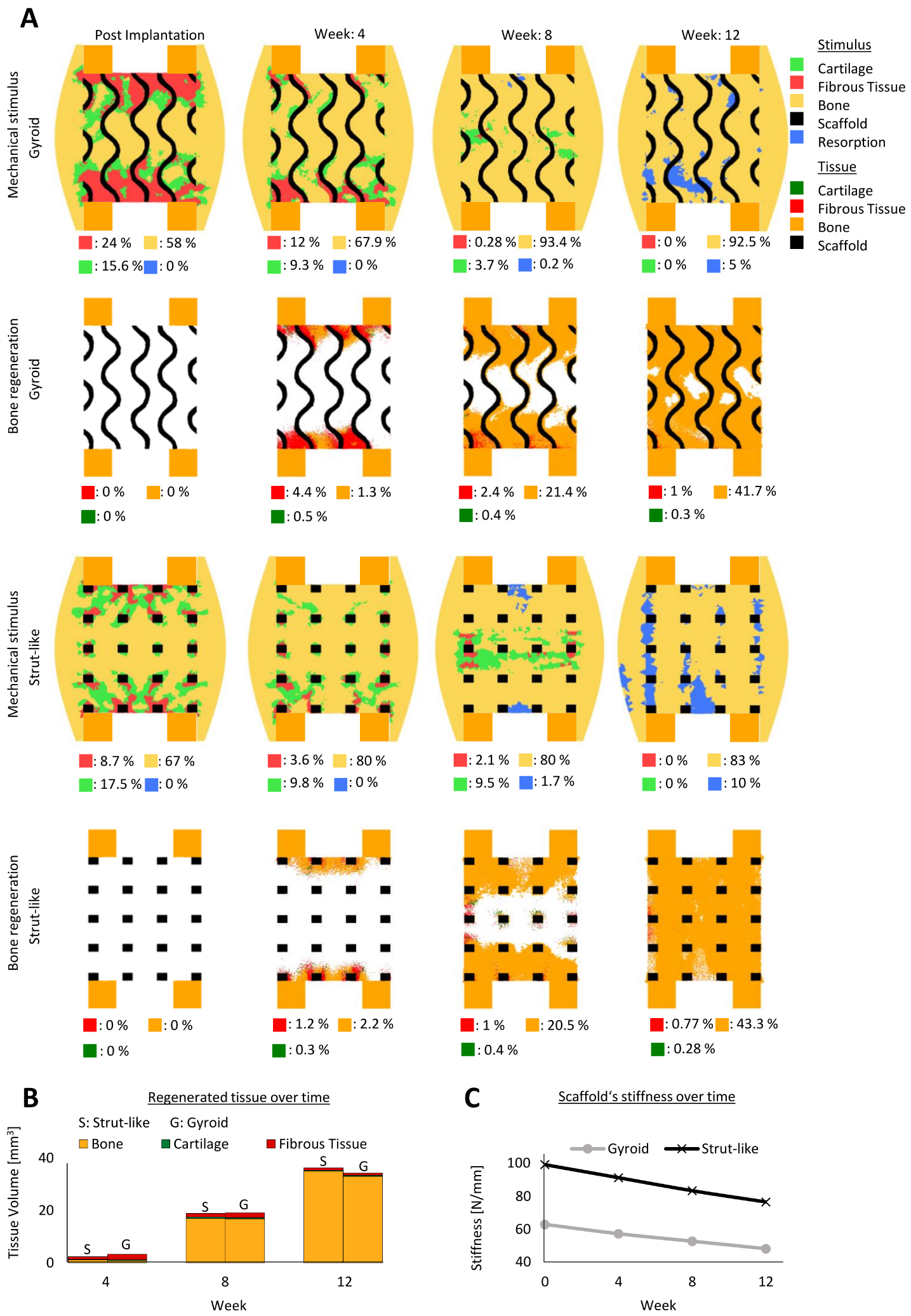


**Supplementary Figure 5.** Longitudinal cross sections of the callus from Figure 1A with quantified tissue regeneration in the 2D section, : The minimum, maximum and maximum absolute principal strains of (A): Strut-like scaffold 79% porosity, (B): Gyroid scaffold 79% porosity, (C): Strut-like scaffold 69% porosity and (D): Gyroid scaffold 69% porosity
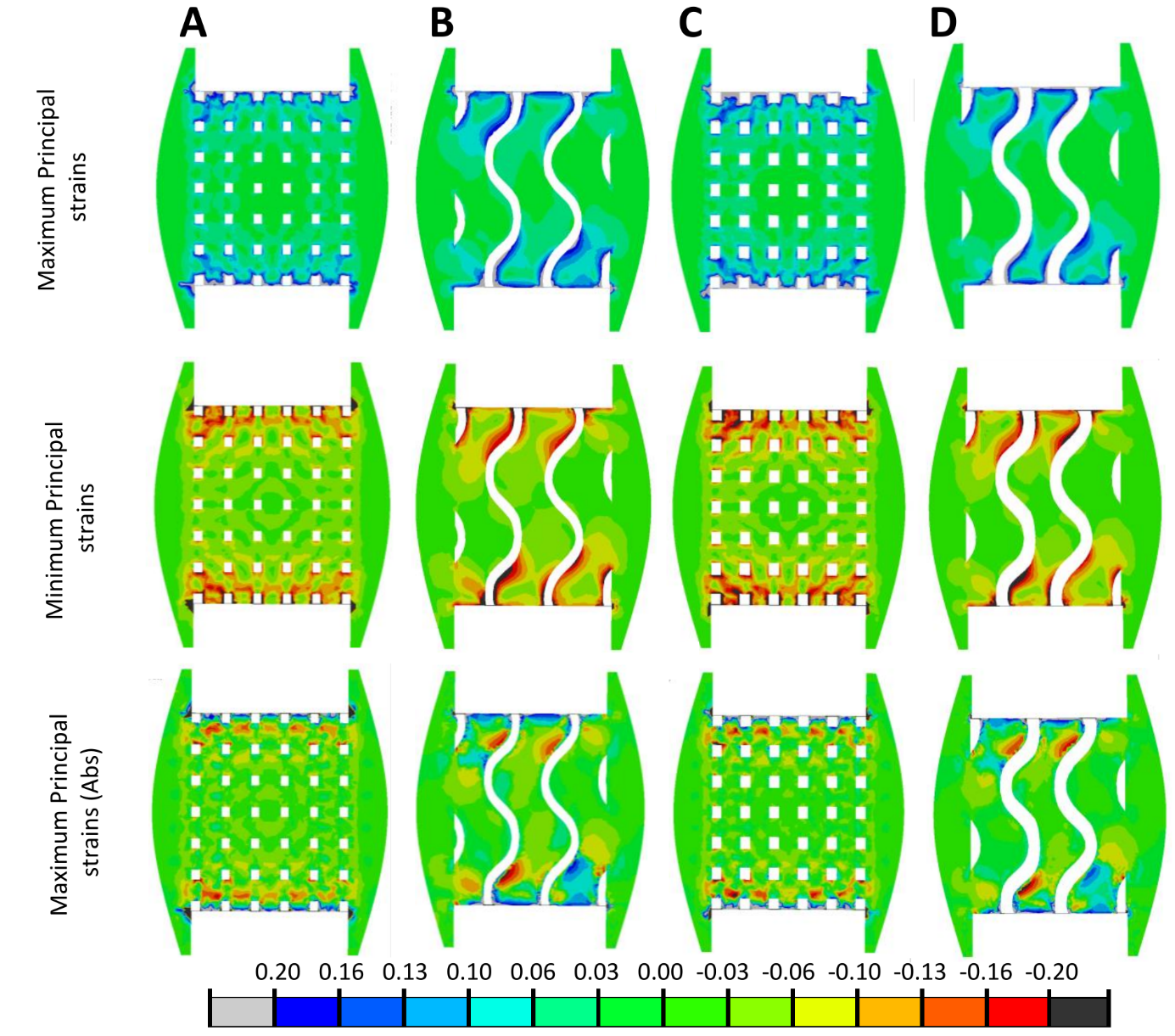


**Supplementary Table 1.** Total number of tissue including bone, cartilage and fibrous tissue quantified in 4, 8 and 12-week time points for several scaffold design of a total volume: 62 ${mm}^{3}$

| Week | 4 weeks | | | 8 weeks | | | 12 weeks | | |
| --- | --- | --- | --- | --- | --- | --- | --- | --- | --- |
| Simulation | Bone [${mm}^{3}$] | Cartilage  [${mm}^{3}$] | Fibrous tissue [${mm}^{3}$] | Bone [${mm}^{3}$] | Cartilage  [${mm}^{3}$] | Fibrous tissue [${mm}^{3}$] | Bone [${mm}^{3}$] | Cartilage  [${mm}^{3}$] | Fibrous tissue [${mm}^{3}$] |
| \| Gyroid P79 \| \| --- \| | 0.57 | 0.37 | 2.08 | 14.3 | 0.64 | 2.8 | 31 | 0.4 | 1.1 |
| \| Strut-like P79 \| \| --- \| | 1.08 | 0.26 | 1.02 | 17.0 | 0.51 | 1.3 | 35 | 0.34 | 0.88 |
| \| Gyroid P69 \| \| --- \| | 0.56 | 0.32 | 1.98 | 12.9 | 0.51 | 2.3 | 27.5 | 0.27 | 0.91 |
| Strut-like P69 | 0.84 | 0.19 | 0.90 | 14.6 | 0.32 | 1.1 | 28.8 | 0.18 | 0.53 |
| Highly degradable | 0.73 | 0.41 | 1.67 | 14.5 | 0.75 | 2.7 | 32.3 | 1.1 | 5 |
| Non degradable | 0.56 | 0.38 | 2.12 | 14.3 | 0.61 | 2.8 | 31 | 0.36 | 1.1 |
